# 硫利达嗪通过内质网应激介导DR5表达上调增敏TRAIL对肺癌PC9细胞的促凋亡效应

**DOI:** 10.3779/j.issn.1009-3419.2017.02.02

**Published:** 2017-02-20

**Authors:** 娟 李, 毅 汪, 柳 刘, 媛 袁, 扬漪 鲍

**Affiliations:** 1 230061 合肥，安徽医科大学第三附属医院（合肥市第一人民医院）肿瘤科 Department of Oncology, the Third Affiliated Hospital of Anhui Medical University, Hefei 230061, China; 2 230061 合肥，合肥市滨湖医院中心实验室 Central Laboratory of Hefei Binhu Hospital, Hefei 230061, China

**Keywords:** 肺肿瘤, 硫利达嗪, TRAIL, 内质网应激, 死亡受体5, Lung neoplasms, Thioridazine, TRAIL, Endoplasmic reticulum stress, DR5

## Abstract

**背景与目的:**

肿瘤坏死因子相关凋亡诱导配体（tumor necrosis factor-related apoptosis-inducting ligand, TRAIL）可诱导肿瘤细胞发生凋亡，然而相当数量的肿瘤细胞可耐受TRAIL诱导的凋亡而得以存活。本实验观察硫利达嗪（thioridazine, THZ）通过诱导内质网应激（endoplasmic reticulum stress, ER stress）介导的死亡受体5（death receptor 5, DR5）表达上调，继而增敏TRAIL对肺腺癌细胞PC9的生长抑制及凋亡诱导效应，探讨其机制。

**方法:**

不同浓度硫利达嗪及TRAIL单独或联合处理PC9细胞，MTT法检测细胞活性变化，流式细胞术检测细胞表面DR5表达及细胞凋亡率，Western blotting检测内质网应激相关蛋白GRP78（glucose regulated protein 78）、C/EBP环磷酸腺苷反应元件结合转录因子同源蛋白（C/EBP homologous protein, CHOP）、p-PERK（PKR-like ER kinase）、p-eIF2α（eukaryotic initiation factor-2α, eIF2α）、ATF4（activating transcription factor 4, ATF4）及凋亡相关蛋白Caspase-3、Caspase-9、Caspase-8、PARP、DR5表达变化。

**结果:**

硫利达嗪对PC9细胞的增殖抑制效应呈浓度依赖性（*P* < 0.05），硫利达嗪可增加TRAIL对PC9细胞的抑制作用及凋亡诱导作用且可上调PC9细胞表面DR5表达水平，流式细胞术结果显示：TRAIL联合硫利达嗪组细胞凋亡率较单独TRAIL组显著增加（*P* < 0.05），Western blotting结果显示：TRAIL联合硫利达嗪组细胞Cleaved-caspase-8、Cleaved-PARP、DR5表达水平较单独TRAIL组明显上调。DR5表达上调及促凋亡效应是通过诱导内质网应激发生，并伴随着GRP78及CHOP表达上调发生的，且该效应可被4-苯基丁酸（4-phenylbutyric acid, 4-PBA）可抑制（*P* < 0.05）。

**结论:**

硫利达嗪增敏TRAIL对PC9细胞的增殖抑制效应显著，其机制可能与硫利达嗪内质网应激介导的DR5上调有关。

## Abstract (en)

**Background and objective:**

Tumor necrosis factor-related apoptosis-inducting ligand (TRAIL) can induce apoptosis of tumor cells, however, various of tumor cells may survive because of resistance to TRAIL-mediated apoptosis. This study is to observe the proliferation inhibition effect of TRAIL sensitized by thioridazine on PC9 cells through endoplasmic reticulum (ER) stress mediated up-regulation of death receptor 5 (DR5) and investigate its mechanism.

**Methods:**

PC9 cells were treated with different concentrations of thioridazine and TRAIL alone or in combination. Cell proliferation was measured by MTT assay, and cell apoptosis and cell-surface DR5 were detected by flow cytometry. Western blotting was utilized to measure the expressions of ER stress-related proteins glucose regulated protein 78 (GRP78), C/EBP homologous protein (CHOP), p-PKR-like ER kinase (PERK), p-eukaryotic initiation factor-2α (eIF2α), activating transcription factor 4 (ATF4) and apoptosis-related proteins caspase-3, caspase-9, caspase-8, PARP, DR5.

**Results:**

Thioridazine inhibited the proliferation of PC9 cells in a dose-dependent manner (*P* < 0.05). Thioridazine increased the inhibition and apoptosis of PC9 cells and upregulated the expression of cell-surface DR5 induced by TRAIL. Flow cytometry showed that compared with TRAIL group, combination group of TRAIL and thioridazine increased cell apoptotic rates significantly (*P* < 0.05). Western blotting indicated that compared with TRAIL group, expressions of Cleaved-caspase-8, Cleaved-PARP and DR5 increased significantly in combination group of TRAIL and thioridazine. The induction of DR5 and pro-apoptotic effect were mediated through activation of ER stress accompanying by increased synthesis of GRP78 and CHOP, which can be blocked by adding of ER stress inhibitor 4-PBA.

**Conclusion:**

Thioridazine enhanced proliferation inhibition effect of TRAIL in PC9 cells may be facilitated through ER stress mediated upregulation of DR5.

肿瘤坏死因子相关凋亡诱导配体（tumor necrosis factor-related apoptosis-inducting ligand, TRAIL）属于TNF超家族，可诱导肿瘤细胞发生凋亡。TRAIL可通过与其受体TRAIL-R1（death receptor 4, DR4）、TRAIL-R2（death receptor 5, DR5）结合促使靶细胞发生凋亡。然而，相当数量的肿瘤细胞可耐受TRAIL诱导的凋亡而得以存活——针对此类已发生TRAIL抵抗的肿瘤细胞，仅以TRAIL处理无法对之进行杀伤。肿瘤细胞对TRAIL发生耐受的原因是多方面的：肿瘤细胞表面的DR4、DR5表达低下或功能异常、TRAIL与DR4、DR5结合后胞内信号转导受阻、胞内抗凋亡蛋白高表达以及促生存通路活化等因素均能使肿瘤细胞对TRAIL发生原发（继发）的抵抗^[1]^。理解肿瘤细胞TRAIL抵抗现象的发生机制有助于逆转肿瘤细胞对TRAIL的耐受；此外，将传统化疗或表观调节类药物（即增敏剂）与TRAIL处理相联合作用于肿瘤细胞，上调TRAIL的促凋亡效应也是目前TRAIL的研究重点之一。TRAIL治疗晚期非小细胞肺癌^[2]^（non-small cell lung cancer, NSCLC）及转移的结直肠癌^[3]^已经进入Ⅱ期临床试验，但肿瘤细胞对TRAIL的抵抗问题同样限制了其在临床上的应用。

硫利达嗪（thioridazine, THZ）为一种多巴胺受体阻滞剂，广泛应用于精神分裂症的治疗，最近其被证实对多种肿瘤细胞具有抗增殖活性^[4-7]^。本课题组前期实验证实^[8]^，THZ可抑制食管癌ECA-109、TE-1细胞的增殖，且对食管癌细胞具有放射处理增敏效应，其机制可能与下调线粒体抗凋亡蛋白Bcl-2、Bcl-xL及抑制PI3K/AKT/mTOR通路活性有关。据统计，我国肺癌患者部分存在表皮生长因子受体（epidermal growth factor receptor, *EGFR*）突变，而在晚期肺腺癌患者中，*EGFR*突变阳性率高达50%左右^[9]^，临床试验发现，针对*EGFR*突变患者的靶向药物——EGFR酪氨酸激酶抑制剂（EGFR tyrosine kinase inhibitors, EGFR-TKIs）如吉非替尼等治疗在取得疗效进展的同时也存在一定程度的耐药问题^[10]^，因此本实验以*EGFR*突变型NSCLC PC9细胞为研究对象，观察THZ对TRAIL的杀伤增敏效应及对TRAIL抵抗的逆转作用，并探讨其可能的作用机制。

## 1 材料与方法

### 1.1 材料

#### 1.1.1 实验细胞株

人肺腺癌细胞株PC9购买于中国科学院上海细胞库。

#### 1.1.2 主要药品与试剂

硫利达嗪，4-PBA购于美国Sigma公司；二甲基亚砜（DMSO）、MTT试剂购于美国Sigma公司；DMEM高糖培养基、1×PBS缓冲溶液购于美国Hyclone公司；胎牛血清购于浙江天杭生物；Annexin V-FITC/PI双染细胞凋亡检测试剂盒购于上海贝博生物公司；胰酶、SDS-PAGE凝胶配制试剂盒购于北京碧云天生物公司；重组人TRAIL蛋白（rh-TRAIL）、DR5-PE流式抗体购于美国eBioscience公司；兔抗人GRP78单抗、p-eIF2α单抗、ATF4单抗、Caspase-3多抗、PARP单抗及小鼠抗人CHOP单抗、Caspase-8单抗、Caspase-9单抗购于美国Cell Signaling Technology公司；兔抗人DR5单抗购于英国Abcam公司；兔抗人p-PERK多抗购于美国Santa Cruz Biotechnology公司；兔二抗及小鼠二抗购于北京中杉金桥公司。

### 1.2 细胞培养与药物保存使用

配制含有10%胎牛血清的DMEM完全培养基，按培养基体积加入双抗，该培养基用于PC9细胞培养与传代。加入培养基后将细胞置于37 ℃、5%CO_2_饱和湿度恒温培养箱中培养。注意观察细胞生长情况及密度，每天全量换液，当细胞呈对数生长时用0.25%胰酶消化传代。THZ以DMSO为溶媒进行配制，浓度为0.1 mol/L，为避免药物的反复冻融，将其分装成每管10 μL于-80 ℃冰箱储存，使用时以完全培养基稀释成需要的浓度，DMSO终浓度须小于0.1%。重组人TRAIL蛋白以去离子水为溶酶进行配制，浓度为0.1 mg/mL，分装为每管10 μL于-20 ℃保存，使用时以完全培养基稀释成需要的浓度。

### 1.3 THZ和TRAIL单药或联合处理PC9细胞MTT法实验步骤

取呈对数生长PC9细胞，按8×10^3^个/孔接种于96孔板中，培养至细胞呈对数生长时，对照组换液，实验组每孔更换含不同浓度的THZ（0 μmol/L、10 μmol/L、20 μmol/L、25 μmol/L、30 μmol/L、35 μmol/L、40 μmol/L、45 μmol/L、50 μmol/L）的培养基，各组均设置3个副孔和一个空白孔，培养24 h。为观察THZ与TRAIL对细胞增殖的影响，细胞给予不同浓度THZ（0 μmol/L、20 μmol/L、25 μmol/L、30 μmol/L）、TRAIL（0 ng/mL、10 ng/mL、50 ng/mL、100 ng/mL）单独或联合处理，培养24 h后应用MTT检测THZ对细胞生长状况的影响，酶标仪（490 nm和655 nm双波长）测定各孔吸光度值（optical density, OD），绝对吸光度值=细胞孔吸光度值-空白孔吸光度值，并计算细胞存活率=（实验组OD值/对照组OD值）×100%。实验重复3次。

### 1.4 Western blotting检测凋亡相关蛋白、内质网应激关键蛋白的表达

取对数生长细胞，按实验分组更换含THZ培养基，实验组1：0 μmol/L（对照组）、25 μmol/L、30 μmol/L、35 μmol/L；实验组2：0 μmol/L（对照组）、25 μmol/L THZ联合50 ng/mL TRAIL、50 ng/mL TRAIL、1 mmol/L 4-PBA及25 μmol/L THZ联合50 ng/mL TRAIL，培养24 h后常规胰酶消化收集细胞，计数后加入相应量的细胞裂解液后沸水浴裂解提取蛋白。蛋白样品离心处理后制胶，上样，电泳，转膜，封闭液室温封闭1 h，室温孵育1:1, 000一抗1 h，1×TBST洗膜10 min，3遍，室温孵育1:5, 000二抗1 h，1×TBST洗膜10 min，3遍，在PVDF膜表面涂布发光剂，暗室曝光显影。Western blotting条带经扫描仪扫描成像后，采用Image J进行灰度分析，计算目的蛋白与β-actin的灰度比值，进行下一步统计分析。实验重复3次。

### 1.5 流式检测细胞表面DR5表达水平

取处于对数生长期的PC9细胞，按2×10^5^个/孔接种于六孔板。常规培养至细胞呈对数生长时，按实验分组更换含培养基，实验组为：THZ（25 μmol/L）、THZ（25 μmol/L）联合4-PBA（1 mmol/L），对照组加0.1%DMSO培养基，继续培养24 h后收集细胞，PBS洗涤后重悬，DR5抗体孵育，混匀，4 ℃反应30 min，上流式细胞仪检测。实验重复3次。

### 1.6 流式检测细胞凋亡率

取处于对数生长期的PC9细胞，按2×10^5^个/孔接种于六孔板。常规培养至细胞呈对数生长时，按实验分组更换含培养基，实验组为：TRAIL（50 ng/mL）、TRAIL（50 ng/mL）联合THZ（25 μmol/L）、4-PBA（1 mmol/L）干预TRAIL（50 ng/mL）联合THZ（25 μmol/L），对照组加培养基，继续培养24 h后收集细胞，PBS洗涤后重悬，计数，加入400 μL Annexin V结合液重悬细胞，调节细胞密度为1×10^6^个/mL，加入5 μL Annexin V，再加入10 μL PI，混匀，室温下避光反应15 min，上流式细胞仪检测细胞凋亡，计算细胞凋亡率。实验重复3次。

### 1.7 统计学方法

采用SPSS 19.0软件进行统计分析。实验数据以Mean±SD表示，组间数据比较采用单因素方差分析及*Dunnett*-*t*检验，显著性检验水准取α=0.05，以*P* < 0.05为差异有统计学意义。

## 2 结果

### 2.1 THZ对PC9细胞的增殖抑制作用

MTT结果显示，THZ对PC9细胞的增殖抑制效应呈剂量依赖性，THZ对PC9细胞的半数抑制浓度值（half maximal inhibitory concentration, IC_50_）为（29.8±1.6）μmol/L。自20 μmol/L起，各实验组与对照组相比差异有统计学意义（*P* < 0.05）（图 1A）。400倍光镜下观察细胞，实验组细胞随着药物浓度增加，细胞数量减少，贴壁减少，体积缩小，边缘透亮，漂浮状细胞增加（图 1B）。Western blotting结果显示随着THZ浓度增加，Cleaved-caspase-3及Cleaved-caspase-9表达水平明显增加（图 1C）。

**1:**
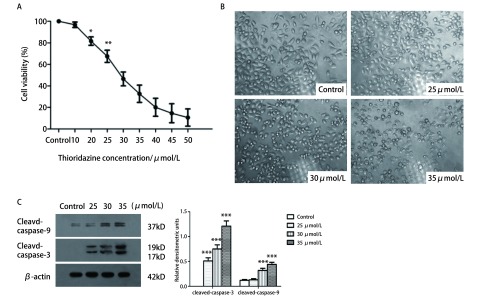
THZ对PC9的增殖抑制作用。A：THZ呈：剂量依赖性抑制PC9细胞增殖。不同浓度THZ（10 *μ*mol/L、20 *μ*mol/L、25 *μ*mol/L、30 *μ*mol/L、35 *μ*mol/ L、40 *μ*mol/L、45 *μ*mol/L和50 *μ*mol/L）处理细胞24 h，MTT检测细胞存活率。B：光镜观察THZ对PC9细胞形态影响（×400）。C：不同浓度THZ（25 *μ*mol/L、30 *μ*mol/L、35 *μ*mol/L）处理细胞24 h后观察细胞形态变化。不同浓度THZ（25 *μ*mol/L、30 *μ*mol/L、35 *μ*mol/L）处理细胞24 h后，Western blotting检测Cleaved-caspase-3及Cleaved-caspase-9表达情况，^*^P < 0.05, ^**^P < 0.01，^***^P < 0.001。 The proliferation inhibition effect of THZ on PC9 cells. A: THZ inhibited cell survival of PC9 cells in a dose-dependent manner. Cells were treated with different concentrations of THZ (10 *μ*mol/L, 20 *μ*mol/L, 25 *μ*mol/L, 30 *μ*mol/L, 35 *μ*mol/L, 40 *μ*mol/L, 45 *μ*mol/L and 50 *μ*mol/ L) for 24 h, and MTT assays was utilized to measure cell viability. B: Morphologic observation of PC9 cells treated with THZ (×400). C: Cells were treated with different concentrations of THZ (25 *μ*mol/L, 30 *μ*mol/L and 35 *μ*mol/L). After treated with different concentration of THZ (25 *μ*mol/ L, 30 *μ*mol/L and 35 *μ*mol/L), expression of Cleaved-caspase-3 and Cleaved-caspase-9 were detected by Western blotting. ^*^P < 0.05, ^**^P < 0.01, ^***^P < 0.001.

### 2.2 MTT检测THZ与TRAIL联合的协同指数

THZ联合TRAIL能显著降低细胞的存活率（图 2），25 μmol/L THZ及50 ng/mL TRAIL联合作用于PC9细胞的CI值为0.623（CI < 1）（图 2），表明THZ及TRAIL可协同抑制PC9细胞存活率。

**2:**
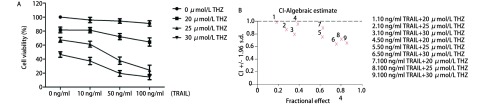
THZ联合TRAIL抑制PC9细胞增殖不同浓度THZ（20 *μ*mol/L、25 *μ*mol/L、30 *μ*mol/L）联合不同浓度TRAIL（10 ng/mL、50 ng/mL、100 ng/mL）作用于细胞24 h后，细胞的存活率（A）及THZ与TRAIL的联合指数（B）。 Combination treatment of THZ and TRAIL inhibited cell survival in PC9 cells. Cells were treated with different dose of THZ (20 *μ*mol/L, 25 *μ*mol/L and 30 *μ*mol/L) and different dose of TRAIL (10 ng/mL, 50 ng/mL and 100 ng/mL) for 24 h. Cell viability (A) was measured by MTT assays and combination index (CI) of THZ and TRAIL (B) were calculated.

### 2.3 THZ诱导PC9细胞发生内质网应激及内质网应激抑制剂4-PBA对细胞表面DR5表达的影响

Western blotting结果显示：不同浓度THZ诱导PC9细胞发生内质网应激，随着THZ浓度增加，内质网应激关键蛋白GRP78、CHOP表达上调（*F*=11.45, *P*=0.001, 4; *F*=12.94, *P* < 0.01），相关蛋白p-PERK（*F*=35.61, *P* < 0.01）、p-eIF2α（*F*=54.26, *P* < 0.01）、ATF4（*F*=38.04, *P* < 0.01）表达上调，与对照组相比，差异有统计学意义。流式细胞术显示：25 μmol/L THZ可显著上调细胞表面DR5表达，而4-PBA可抑制THZ对细胞表面DR5的上调，差异有统计学意义（*F*=61.19, *P* < 0.01）（图 3）。

**3:**
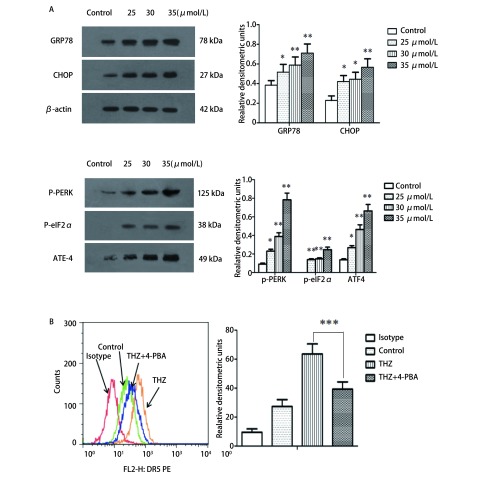
THZ诱导PC9细胞发生内质网应激。A：THZ诱导细胞内质网应激相关蛋白表达变化，不同浓度THZ（25 *μ*mol/L、30 *μ*mol/L、35 *μ*mol/L）作用于细胞24 h后，Western blotting检测GRP78、CHOP、p-PERK、p-eIF2α、ATF4表达水平；B：流式细胞术检测THZ（25 *μ*mol/L）单独或联合4-PBA（1 mmol/L）处理PC9细胞对细胞表面DR5表达的影响。THZ作用于细胞24 h可上调细胞表面DR5表达，而4-PBA联合THZ组可显著抑制细胞DR5表达上调。^*^P < 0.05, ^**^P < 0.01, ^***^P < 0.001。 ER stress induced by THZ in PC9 cells. A: Expression of ER stress related proteins induced by THZ; B: Cell surface DR5 was detected by flow cytometry of PC9 cells following THZ (25 *μ*mol/L) or combined with 4-PBA (1 mmol/L). After treated with thioridazine 24 h, cell surface DR5 increased significantly in PC9 cells, which can be inhibited by 4-PBA. ^*^P < 0.05, ^**^P < 0.01, ^***^P < 0.001.

### 2.4 THZ可增敏TRAIL对PC9细胞的杀伤效应且4-PBA可抑制此效应

Western blotting结果显示：THZ联合TRAIL可显著增加Cleaved-caspase-8、Cleaved-PARP及DR5表达水平（图 4A）。流式细胞术结果显示：THZ联合TRAIL组细胞凋亡率为（32.6±4.7）%，单TRAIL处理组为（3.6±1.3）%，加入了4-PBA的THZ联合TRAIL组为（17.3±2.9）%，与对照组及单独TRAIL组相比，THZ联合TRAIL组细胞凋亡率明显增加，而4-PBA可显著抑制THZ增敏TRAIL诱导的细胞凋亡，与THZ联合差异有统计学意义（*F*=93.51, *P* < 0.01）（图 4B）。

**4:**
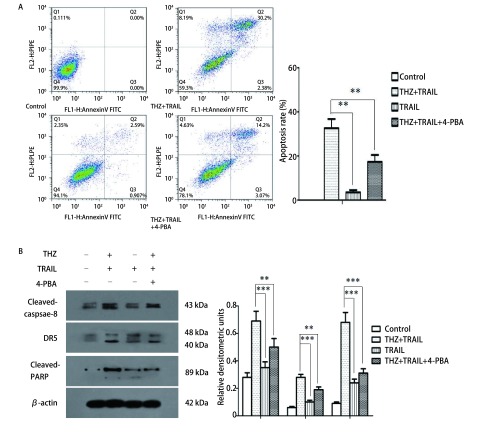
THZ联合TRAIL诱导PC9细胞凋亡。A：流式细胞术检测THZ（25 *μ*mol/L）联合TRAIL（50 ng/mL）组、TRAIL组、4-PBA（1 mmol/L）及THZ联合TRAIL组细胞凋亡率；B：Western blotting分析各组Cleaved-caspase-8、Cleaved-PARP及DR5表达水平。^**^P < 0.01, ^***^P < 0.001。 Cell apoptosis induced by combination treatment of THZ and TRAIL in PC9 cells. Cells were treated with TRAIL (50 ng/mL) and/or THZ (25 *μ*mol/L) and/or 4-PBA (1 mmol/L) for 24 h. The apoptosis rate (A) and expression of Cleaved-caspase-8, Cleaved-PARP and DR5 (B) of PC9 cells were measured by flow cytometry and Western blotting respectively. ^**^P < 0.01, ^***^P < 0.001.

## 3 讨论

自2012年Burgess及Sachlos等发现THZ可显著降低人类急性髓细胞白血病（acute myeloid leukemia, AML）细胞的增殖与自我更新能力以来^[11, 12]^，THZ陆续被证实在体内外对多种肿瘤细胞具有抗增殖活性——包括宫颈癌细胞、子宫内膜癌细胞^[4]^、卵巢癌细胞^[5]^、乳腺癌细胞^[6]^、胃癌细胞^[7]^等。本研究组之前证实15 μmol/L THZ即可显著诱导食管癌细胞凋亡及周期阻滞，且可在体内外增敏放疗的杀伤效应，其机制可能与下调线粒体抗凋亡蛋白Bcl-2、Bcl-xL及抑制PI3K/mTOR通路的激活有关^[8]^。THZ可能作为一种抗肿瘤的新药应用于今后的临床治疗。

THZ单独处理对PC9细胞具有明显的杀伤效应，表现为Cleaved-caspase-3、Cleaved-caspase-9表达水平上升，提示THZ具有显著的体外抗肿瘤效应——这与课题组之前结果一致^[13]^。缺血再灌注损伤、氧化应激、药物及放射线处理等因素均可导致内质网应激发生，由此产生的未（错误）折叠的蛋白堆积、细胞蛋白质合成减少及内质网降解功能增强等级联反应，称为未折叠蛋白反应（unfolded protein response, UPR）^[14]^。若内质网应激持续时相过长或是强度过高，且应激因素无法消除时，细胞将因无法及时去除错误折叠蛋白而最终启动凋亡途径^[15]^。内质网应激反应为双向调节反应，低程度的内质网应激上调细胞适应能力，保护细胞免于凋亡——在肿瘤治疗中可表现为肿瘤细胞对化疗药物的抵抗性升高，参与化疗耐药的发生过程。UPR主要由GRP78介导，当细胞发生内质网应激时，感受器IRE1、PERK以及ATF6可与GRP78解离并进一步激活各自的信号通路^[16]^，故GRP78是内质网应激发生的标志性分子。其中，活化的PERK使eIF2α磷酸化，磷酸化的eIF2α能下调胞内蛋白质的合成，但能增加ATF4的转录表达，ATF4表达能进一步诱导CHOP表达，该通路为CHOP表达所必需的通路^[17]^，而CHOP被认为是内质网应激介导的凋亡中的关键分子，GRP78的过表达将会抑制CHOP的表达及凋亡的发生。研究^[18]^表明，抑制PERK/eIF2α通路可显著增强细胞凋亡作用。本实验发现，25 μmol/L THZ处理PC9细胞后GRP78、CHOP表达水平比对照组明显增加，且随着药物浓度增加，其表达水平逐渐增加，提示THZ处理可使PC9细胞发生内质网应激，同时PC9细胞内的p-PERK、p-eIF2α、ATF4表达水平较对照组明显上调，PERK/eIF2α通路被显著激活。最终上调CHOP表达，使PC9凋亡过程启动。

TRAIL可表达于包括活化及静息B淋巴细胞、活化的T细胞、NK细胞、单核细胞、巨噬细胞及树突状细胞在内的多种免疫细胞，是其重要杀伤性配体，TRAIL单体蛋白也被证实可诱导肿瘤细胞发生凋亡且，对正常细胞（组织）毒性较低。TRAIL与其受体DR4/DR5结合后可形成死亡诱导信号复合体（death inducing signaling complex, DISC），依次激活caspase-8/10、caspase-3启动凋亡发生——经上述途径凋亡的细胞称为Ⅰ型细胞。而在某些细胞中，TRAIL与DR4或DR5结合后诱导的外源性促凋亡通路尚不足以启动靶细胞凋亡，尚需线粒体途径的参与，称为Ⅱ型细胞^[19]^。肿瘤细胞可对TRAIL诱导的凋亡发生抵抗，其机制可能与其高表达诱骗受体（Decoy receptors）继而竞争性抑制DR4和/或DR5有关。此外，肿瘤细胞DR4和/或DR5表达缺失也是TRAIL抵抗发生的重要因素。耐受TRAIL的肿瘤细胞在体外能被化疗药物增敏，提示联合治疗在处理TRAIL抵抗肿瘤细胞中可能有一定的意义。研究表明，乳腺癌细胞株发生TRAIL抵抗与其细胞膜表面DR4/DR5内吞，导致乳腺癌细胞株表面DR4、DR5缺如关系密切^[20]^。槲皮素可通过上调DR5增敏TRAIL诱导卵巢癌细胞凋亡^[21]^，TRAIL抵抗肿瘤细胞中DR5过表达可重塑TRAIL的敏感性^[22]^。本实验流式结果显示，50 ng/mL TRAIL与25 μmol/L THZ联合处理可显著降低细胞存活率，两药具有协同作用，联合组细胞凋亡率明显增加。其原因可能与THZ显著增加PC9细胞表面DR5表达水平有关。当内质网应激抑制剂4-PBA加入后，THZ诱导的DR5表达上调即被终止，继而降低TRAIL联合THZ处理组细胞的凋亡率。提示THZ协同增效TRAIL可能是通过激活ER stress而上调PC9表面DR5表达引起的。Western blotting结果也显示，TRAIL与THZ联合组细胞Cleaved-caspase-8、Cleaved-PARP、DR5表达水平较对照组明显增加，而4-PBA的加入可抑制以上蛋白表达。

综上所述，THZ能诱导人肺腺癌PC9细胞发生内质网应激，并介导DR5上调，继而增敏TRAIL对PC9细胞的凋亡诱导效应，因此THZ可能作为一种有潜力的TRAIL增敏剂。本实验为体外实验，需进一步在体内验证体外实验结果涉及的分子机制。
